# RNA-Seq-Based Profiling of *pl* Mutant Reveals Transcriptional Regulation of Anthocyanin Biosynthesis in Rice (*Oryza sativa* L.)

**DOI:** 10.3390/ijms22189787

**Published:** 2021-09-10

**Authors:** Ruonan Xu, Ronghui Pan, Yuchan Zhang, Yanlei Feng, Ujjal Kumar Nath, Yinbo Gan, Chunhai Shi, Delara Akhter

**Affiliations:** 1 Department of Agronomy, Zhejiang University, Hangzhou 310058, China; xrn0515@zju.edu.cn (R.X.); panr@zju.edu.cn (R.P.); 11716038@zju.edu.cn (Y.Z.); ygan@zju.edu.cn (Y.G.); 2 School of Life Sciences, Westlake University, Hangzhou 310024, China; fengyanlei@westlake.edu.cn; 3 Westlake Institute for Advanced Study, Hangzhou 310024, China; 4 Department of Genetics and Plant Breeding, Bangladesh Agricultural University, Mymensingh 2202, Bangladesh; ujjalnath@gmail.com; 5 Department of Genetics and Plant Breeding, Sylhet Agricultural University, Sylhet 3100, Bangladesh

**Keywords:** *pl* (*purple leaf*) mutant, anthocyanin biosynthesis, transcriptional analysis, DEGs, rice (*Oryza sativa* L.)

## Abstract

Purple-colored leaves in plants attain much interest for their important biological functions and could be a potential source of phenotypic marker in selecting individuals in breeding. The transcriptional profiling helps to precisely identify mechanisms of leaf pigmentation in crop plants. In this study, two genetically unlike rice genotypes, the mutant *purple leaf* (*pl*) and wild (WT) were selected for RNA-sequencing and identifying the differentially expressed genes (DEGs) that are regulating purple leaf color. In total, 609 DEGs were identified, of which 513 and 96 genes were up- and down-regulated, respectively. The identified DEGs are categorized into metabolic process, carboxylic acid biosynthesis, phenylpropanoids, and phenylpropanoid biosynthesis process enrichment by GO analysis. Kyoto Encyclopedia of Genes and Genomes (KEGG) confirmed their association with phenylpropanoid synthesis, flavonoid synthesis, and phenylalanine metabolism. To explore molecular mechanism of purple leaf color, a set of anthocyanin biosynthetic and regulatory gene expression patterns were checked by qPCR. We found that *OsPAL* (*Os02g0626100*, *Os02g0626400*, *Os04g0518400*, *Os05g0427400* and *Os02g0627100*), *OsF3H* (*Os03g0122300*), *OsC4HL* (*Os05g0320700*), and *Os4CL5* (*Os08g0448000*) are associated with anthocyanin biosynthesis, and they were up-regulated in *pl* leaves. Two members of regulatory *MYB* genes (*OsMYB55*; *Os05g0553400* and *Os08g0428200*), two *bHLH* genes (*Os01g0196300* and *Os04g0300600*), and two WD40 genes (*Os11g0132700* and *Os11g0610700*) also showed up-regulation in *pl* mutant. These genes might have significant and vital roles in *pl* leaf coloration and could provide reference materials for further experimentation to confirm the molecular mechanisms of anthocyanin biosynthesis in rice.

## 1. Introduction

The leaf color of the plant is considered to be one of the important morphological traits that might be the potential source of a reliable phenotypic marker in the breeding program [1]. Morphological traits provide a significant contribution to genetic advancement in rice breeding; thus, breeders are looking for the distinct phenotypes that could be used as a marker for effective selection [2,3]. Mechanisms of purple leaf color in plants are quite complex; factors such as the type of pigment as well as their concentration and distribution in leaf cells contribute to leaf colors. Three types of pigment are found in the leaves of the higher plants: (i) chlorophyll (Chl) (mainly Chla and Chlb), (ii) carotenoids (lutein and carotenoid), and (iii) flavonoids (anthocyanins) [4]. Anthocyanins are water-soluble colors and extensively disseminated among the higher plants. They contribute to physiological functions of plants and act as attractants of pollinators and seed dispersers. Recent research has confirmed that anthocyanins have protective functions against different biotic and abiotic stresses [5,6,7,8,9]. It produces coloration of the leaves and leaf sheaths in rice [10]. However, to date, detailed research on the genes involved in anthocyanin biosynthesis in rice has not been carried out. 

The anthocyanin biosynthesis pathway starts from phenylalanine under synchronized regulation of structural genes in the cytoplasm, which encode some regulatory transcription factors and enzymes [11]. Glutathione S-transferases export the anthocyanins from cytoplasm to the vacuole, and they are stored there permanently [12]. The anthocyanin biosynthesis process involves three consecutive stages: firstly, phenylalanine is transformed into transcinnamic acid and 4CL (4-coumarate-CoA ligase), respectively [4,8,13]; thereafter, CHS (chalcone synthase), CHI (chalcone isomerase), and F3H (flavanone-3-hydroxylase) are catalyzed a series of enzymatic reactions and formed dihydroflavonol from one molecule of coumarate-CoA and three molecules of malonyl-CoA; finally, anthocyanidins are produced from dihydroflavonols via catalyzing of DFR (dihydroflavonol 4-reduatse) and ANS (anthocyanidin synthase). These anthocyanidins are modified for glycosylation and methylation to produce anthocyanins through the action of UFGT (UDP glucose: flavonoid glucosyltransferase) and MT (methyl transferase) [14,15]. Earlier studies proved that the expression of the structural genes is regulated by synchronized interactions of R2R3-MYB (myeloblastosis family), WD40-repeat transcription factors, and bHLH (basic helix–loop–helix) [16]. MBW complex (R2R3-MYB, WD40, and bHLH) is very important to plants; its transcriptional regulatory system might be different between mono- and dicotyledonous plants, and all members of this MBW complex are not obligatory for anthocyanin biosynthesis in all species [14,15].

Transcriptome analysis is a dynamic way to detect leaf color genes in any plant species. Therefore, RNA-Seq (RNA sequencing) is used for profiling the transcripts with gene annotation and identification in different plant species [17,18,19]. In this study, RNA-seq was performed by using the leaf of *pl* mutant and its wild type (WT; Zhenong 34) at the grain filling stage. This *pl* mutant was developed by EMS (ethyl methane sulfonate) mutagenesis. It was phenotypically characterized in M_2_ population [20]. We executed transcriptomic analysis of *pl* vs. WT to identify the DEGs for explaining the mechanisms involved in leaf coloration in rice. 

## 2. Results

### 2.1. Phenotypic Characterization 

**Figure 1:**
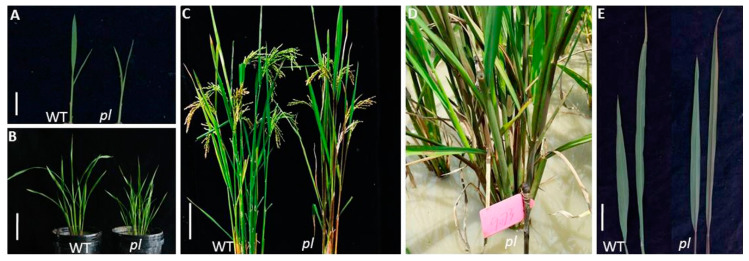
Phenotypic features of WT and pl mutant rice plants. Plants at (**A**) seedling stage, bar: 2 cm; (**B**) tillering stage, bar: 10 cm; (**C**) grain filling stage, bar: 10 cm; (**D**) pl plants showing purple color at field condition; (**E**) magnification of flag leaf from the top of the plant, bar = 5 cm.

The leaf color of *pl* mutant was found to be purple at the grain filling stage, which was not detected in the seedling to tillering stages. The *pl* plants attained a purple color at the grain filling stage (Figure 1A–C), whereas WT remained green (Figure 1D,E). In *pl* mutant, purple coloration started firstly in the leaf blade and midrib, then gradually covered the entire leaf. Additionally, *pl* plants produced shorter grains with fewer numbers of grains/panicle and a lower weight per 1000 grains compared to WT.

### 2.2. RNA-Sequence Analysis

RNA-seq was performed using leaf samples of *pl* mutant and WT plants for profiling the transcriptome. Illumina technology was used to sequence the cDNA of *pl* mutant and WT to identify the possible DEGs and related pathways that are involved in purple leaf coloration. A total of 54,237,834 bp and 55,160,750 bp raw reads were extracted from *pl* and WT samples, respectively (Table 1). After eliminating the adaptor and poor-quality sequences, in total, 52,941,300 bp and 53,875,169 bp clean reads were retrieved from pl and WT samples, respectively, with an average length of 142.99 and 144.14 bp. The average GC content varied from 57.71% to 57.83% in different libraries with >97% Q30 (Table 1).

In total, 106,816,469 clean reads were attained from six cDNA libraries, and they were categorized into three different classes: uniquely mapped, multiple mapped, and total mapped. From the total clean read, 84.95–86.70 % was totally mapped, 77.99–82.21% was uniquely mapped, and 2–6% was multiple mapped (Table 2). 

**Figure 2:**
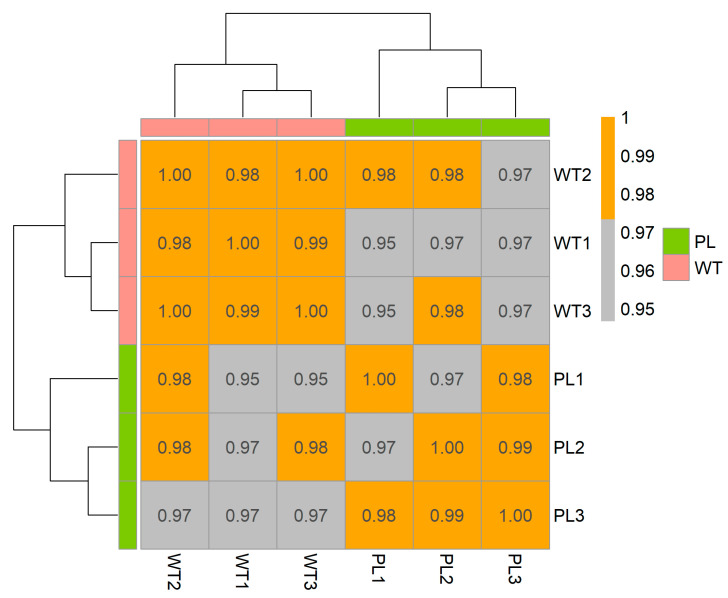
Correlation coefficient using inter-sample RNA-seq values (normalized reads) presented as heat map. Color blocks indicate the correlation coefficient values. The gray color indicates a lower correlation and the orange color represents a higher correlation coefficient between samples.

In the heat map of correlation coefficients of the samples of *pl* mutant and WT (Figure 2), *pl* samples had 0.98 (*pl1*, *pl2*, and *pl3*) average correlation coefficient while WT (WT1, WT2, and WT3) had 0.96, indicating their vicinity toward the unity. 

We found 59,358 and 58,361 SNPs as well as 5,648 and 5,297 indels in *pl* and WT, respectively. SNP regions were separated as inter-genic and genic regions (UTR and CDS), where most of the SNPs were found in the genic regions of the *pl* and WT samples (Table S1). Two types of nucleotide substitutions—transversion (A/T, A/C, G/A C/G and G/T) and transition (A/G, G/A, T/C and C/T)—were found in homozygote SNPs. We found more transition than transversion substitution of SNPs in both *pl* and WT sequences (Table S2).

### 2.3. DEGs Identification

**Figure 3:**
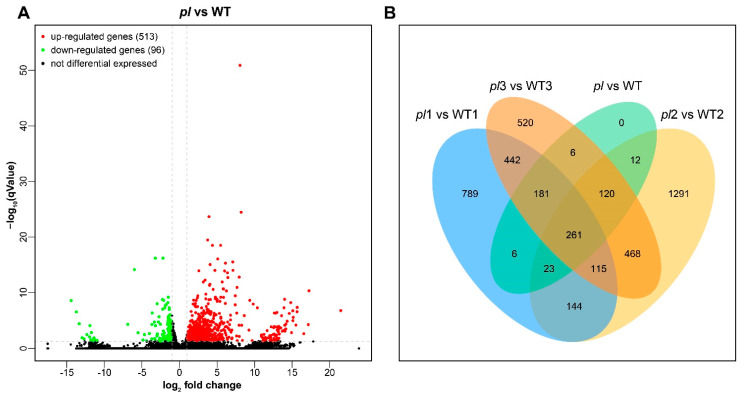
Volcano plot and Venn diagram of DEGs. (**A**) DEGs are presented by volcano map for *pl* vs. WT. Horizontal axis shows fold-change value (log_2_(B/A)) of the differential expression of the genes in different sample groups, whereas the vertical axis shows the level of significance with *p* value depicting the changes of gene expression. Every dot in the figure denotes a gene: green as down-regulation, red as up-regulation, and black as non-differentiated genes. (**B**) DEGs for *pl* vs. WT at grain filling stage are plotted in Venn diagram. Comparisons between different groups are characterized by different colors; numbers in the figure signify the number of shared/specific DEGs. The overlapping regions show the number of shared DEGs by various sets and the non-overlapped regions show the number of unique DEGs.

The DEGs *pl* vs. WT were identified and it was predicted that the genes are involved in the purple leaf color of *pl* mutant (Table S3). The Volcano plot shows the distribution of DEGs at the threshold level for selection. Among 609 DEGs, 513 and 96 were up- and down-regulated in *pl* vs. WT, respectively (Figure 3A, Table S4). In the Venn diagram, a total of 261 genes were identified as commonly shared for all samples (Figure 3B). 

### 2.4. GO Analysis

**Figure 4:**
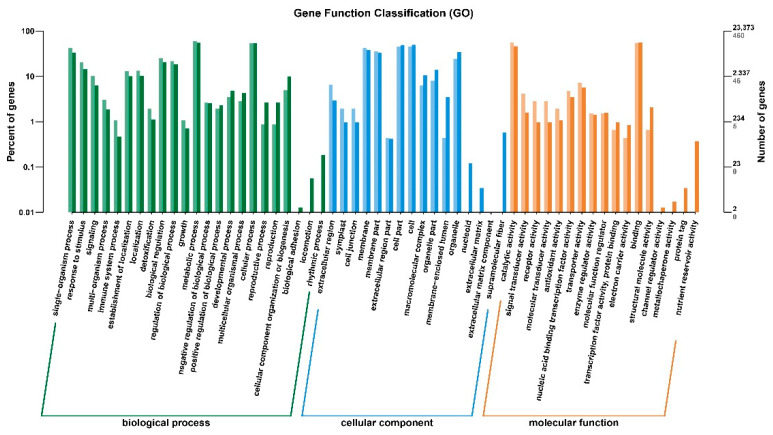
GO analysis of the functional enrichments of down- and up-regulated DEGs between *pl* vs. WT at late grain filling stage. Here, horizontal axis represents functional category and vertical axis represents the gene number in the category (right) and the percentage of the total genes on the annotation (left). Various colors signify various categories. Light color on the axis and histogram represents the differentially expressed genes, and dark color represents total number of genes in the respective categories.

GO (gene ontology) is a global gene function classification that categorizes genes based on their biological processes, cellular components, and molecular functions. Among the DEGs, 40.35% were classified based on their biological processes, 29.82% based on their molecular functions, and 26.32% based on their cellular functions (Figure 4 and Table S5). The majority of the genes in the biological process category were sub-categorized into metabolic process; the rest of the genes were included into the cellular process and single-organism subcategories. A significant proportion of the genes was classified based on catalytic and binding activity. The majority of this portion of genes was included in the cell and cell part followed by membrane, membrane part, and organelle sub-categories (Figure 4). The up- and down-regulated DEGs were categorized according to enriched GO terms by bar plot. A total of 23,373 genes were allotted in GO terms and only 460 (1.96%) DEGs were confirmed as annotated in the database, of which 407 genes were up- and 53 were down-regulated. The scattered plot was constructed by using up- and down-regulated DEGs, which exhibited that most of the genes are involved in metabolic process and rest of them are involved in carboxylic acid and phenylpropanoid biosynthesis processes (Figure S1 and Table S6).

### 2.5. Analysis of DEGs via KEGG and KOG Pathway 

**Figure 5:**
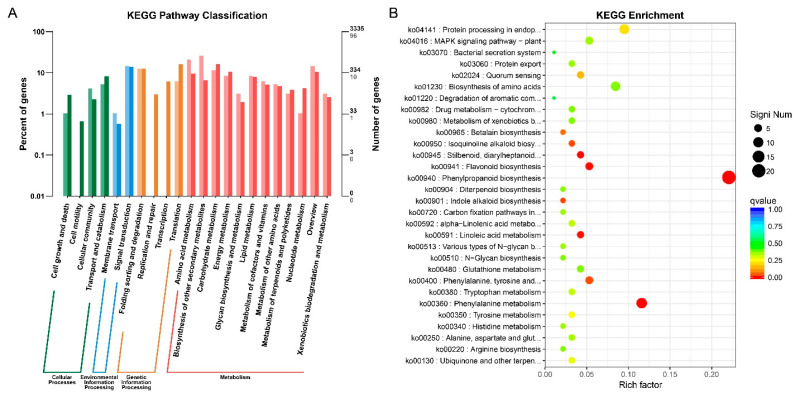
KEGG analysis of DEGs for *pl* vs. WT by bar plot and scattered diagram. (**A**) DEGs analysis by bar plot. Horizontal and vertical axes represent functional category and number of genes in the category (right) and percentage of total genes on the annotation (left), respectively. Colors represent different categories of KEGG enrichment. Light color in the histogram represents the differentially expressed genes and dark color represents total number of genes. (**B**) Scattered plot represents the important enrichment functions. Horizontal axis represents the rich factor function, and the vertical axis represents functional annotation. Top 30 GOs were used for analyzing the Q value, where size is shown by dot color and the smaller Q value is close to red.

The KEGG (Kyoto Encyclopedia of Genes and Genomes) database was implemented to explore the pathways involved in leaf coloration of the *pl* mutant at the late grain filling stage. Bar-plot analysis in the KEGG database was used to identify the uni-gene positioning of metabolic pathways. Only 96 out of 3,335 assigned genes were found to be involved in four pathways: cellular processes, environmental, genetic information processing, and metabolism (Figure 5A and Table S7).

The scattered plot analysis using the top 30 KEGGs showed that the DEGs designated as ko00940 (phenylpropanoid biosynthesis), ko00360 (phenylalanine metabolism), ko00941 (flavonoid biosynthesis), and ko00400 (phenylalanine, tyrosine, and tryptophan biosynthesis) were involved in the leaf coloration-related pathway. The uni-gene positioning was explored through the bar-plot analysis, and it was found that only 96 genes were involved in metabolic, cellular, genetic, and environmental information processing pathways (Figure 5B and Table S8). 

**Figure 6:**
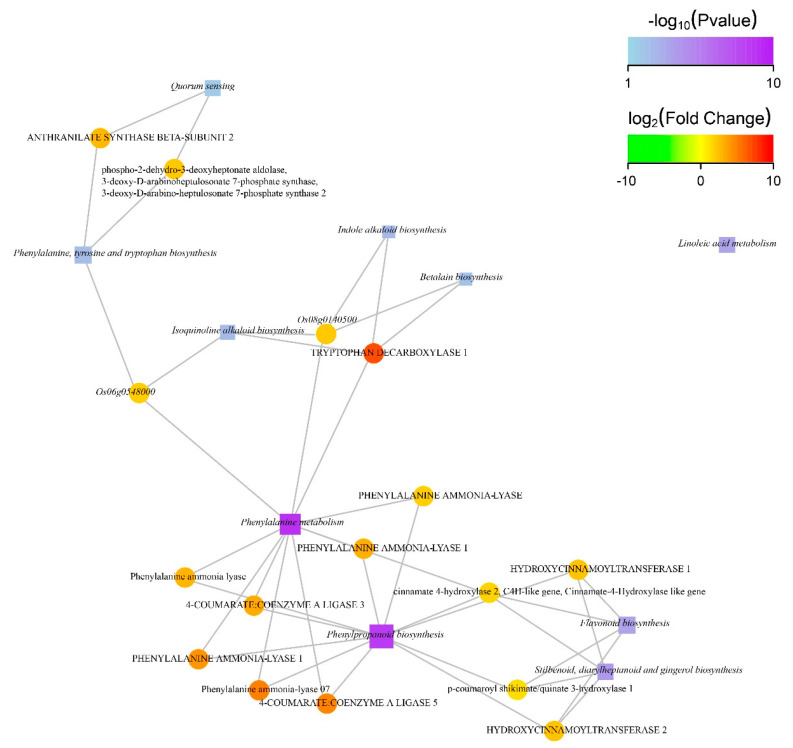
Significantly enriched functions of gene interaction network in KEGG. Square, circular, and edges nodes represent the function information, genes, and correlation between genes and their functions, respectively. The node size is proportional to the degree of connectivity of the nodes; therefore, more edges are connected to the larger node. The color of the circular node represents degree of difference in gene expression. In the log_2_FC value, green and red color represent down- and upward adjustment, respectively, and the intensity of color indicates degree of difference between up- and downward adjustments. The color of the square node represents the *p* value of enrichment program of the function. High enrichment corresponds to low *p* value with dark color. Square node with comparatively larger area indicates a greater number of DEGs involved in the biological phenomena. DEGs related to this function are drawn by taking only top 10 functions with the highest degree of enrichment.

A gene interaction network diagram was generated to identify the involvement of DEGs and their contribution to specific biological phenomena. Most of the genes were found in phenylalanine metabolism, phenylpropanoid biosynthesis, flavonoid biosynthesis, and stilbenoid, diarylheptanoid with gingerol biosynthesis pathways (Figure 6 and Table S8). 

**Figure 7:**
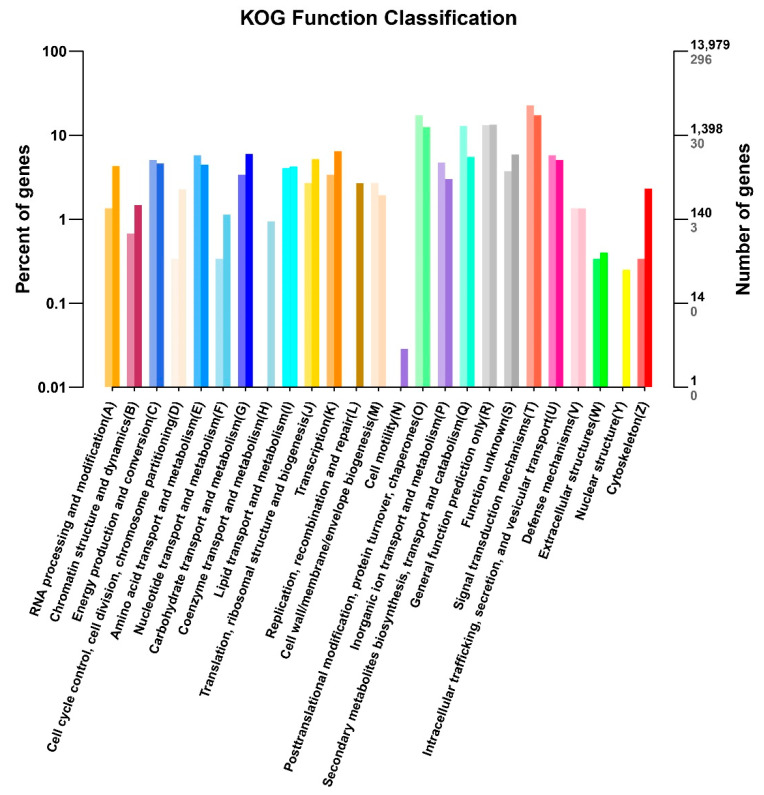
COG/KOG analysis of the DEGs of *pl* vs. WT. Horizontal axis represents the functional category and number of genes (right) and vertical axis represents percentage of the total genes and the total number of DEGs (left). Different categories of DEGs are represented with a wide range of color. Light color in the histogram represents the DEGs and dark color represents total gene number of that category.

Leaf coloration of *pl* mutant might be predicted through the analysis of enrichment. The function of the genes was grouped using the information available in the COG/KOG (Clusters of Orthologous Groups) database based on their orthologs. A total of 13,979 uni-genes were identified as non-redundant in the KOG; among them, 296 genes were differentially expressed. We classified all of the identified uni- and special DEGs into 26 sub-functional groups (Figure 7, Table S9). Maximum genes were found to involve in signal transduction system; thereafter, the rest of the DEGs were found in protein transporter, general post-translational modification, chaperon, secondary metabolite biosynthesis, transport, catabolism, and general function prediction and cell motility.

### 2.6. Validation of the RNA-Seq Data by qRT-PCR

RNA-seq data was validated through qRT-PCR (quantitative real-time polymerase chain reaction) considering 20 randomly selected genes related to purple color in plants (Table 3). Expression pattern of the selected genes showed consistency in sense of up- and down-regulation with RNA-seq data, indicating reliability of the transcriptomes (Table 3). The *pl* mutant had colored leaves with four- and eight-times higher anthocyanin content in the leaf and leaf sheath, respectively, than WT (Table S10). In *pl* mutant, we identified the DEGs related to anthocyanin pathway, which are enriched with MYB, bHLH, and WD40 transcription factors. We also identified 17 DEGs related to the phenylpropanoid biosynthesis rice gene’s ortholog. Among them, six DEGs are related to flavonoid biosynthesis and seven DEGs to the UDP transferase family were up-regulated significantly in *pl* mutant compared to WT (Table 3), which supported our RNA-seq data.

### 2.7. Involvement of Transcription Factors and Anthocyanin Biosynthesis in pl Mutant 

**Figure 8:**
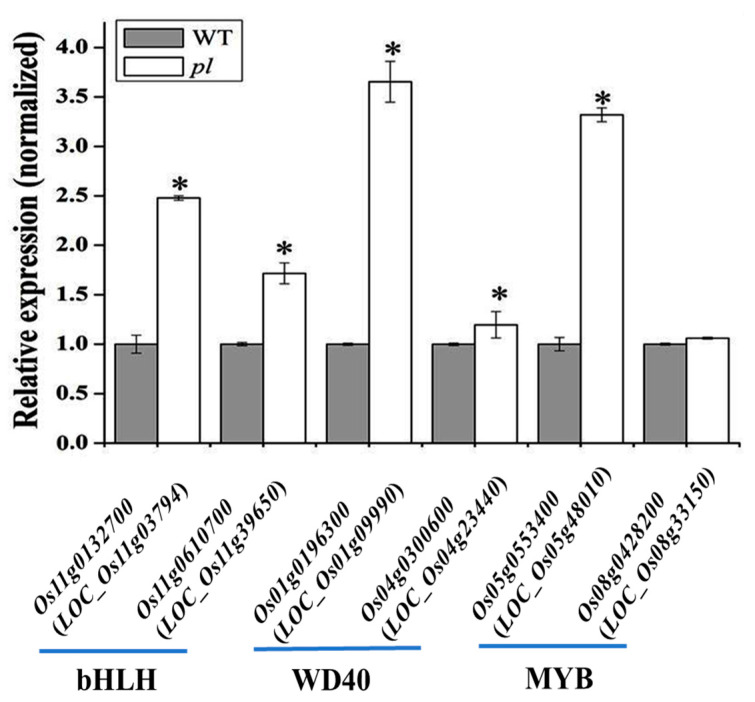
Expression of the transcription factor genes in anthocyanin biosynthesis in *pl* leaf as compared to the WT leaves. * indicates significant difference at *p ≤* 0.05.

The transcription factor families—MYB, WD40, and bHLH—were found to be involved in the anthocyanin biosynthetic process in *pl* mutant. Four DEGs of each of the MYB, bHLH, and WD40 family were identified in RNA-seq data (Table 3 and Figure 8). Candidate transcription factors were screened out through quantification of relative expression by qRT-PCR. Among them, two MYB genes (*Os05g0553400* (*OsMYB55*) and *Os08g0428200*), two bHLH family genes (*Os01g0196300* and *Os04g0300600*), and two WD40 family genes (*Os11g0132700* and *Os11g0610700*) showed up-regulation in *pl* mutant. These genes might have vital roles in establishing the purple leaf color in *pl* mutant and could provide information on anthocyanin biosynthesis mechanisms in rice.

### 2.8. Expression Analysis of Anthocyanin Biosynthesis Genes in pl Mutant 

**Figure 9:**
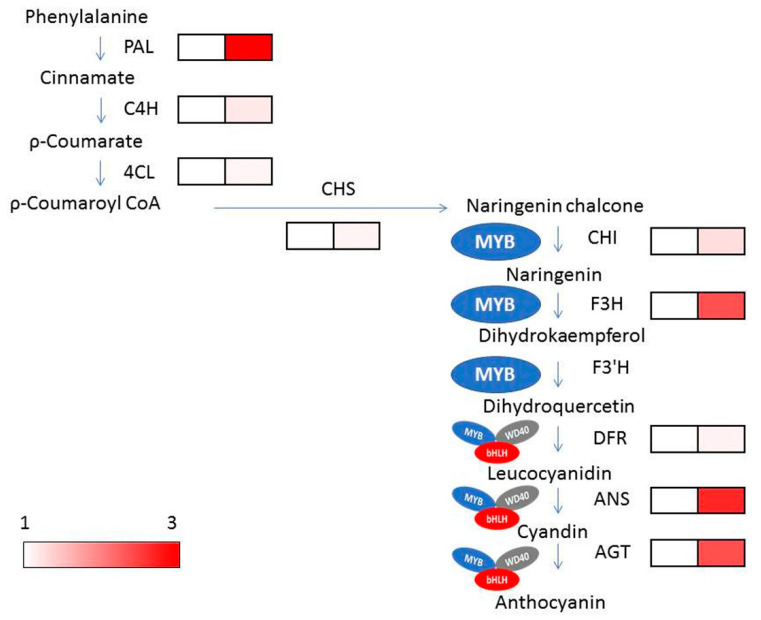
Relative expression patterns of the early and late-structural genes of anthocyanin biosynthesis in leaf samples of *pl* and WT. Colored scales indicate the levels of gene expression.

We found some structural and early biosynthesis genes; *OsPAL* (*Os02g0626100*, *Os02g0626400*, *Os04g0518400*, *Os05g0427400* and *Os02g0627100*), *OsF3H* (*Os03g0122300*), *OsC4HL* (*Os05g0320700*) and *Os4CL5* (*Os08g0448000*) were highly upregulated in *pl* mutant than WT (Figure 9). Out of two MYB genes, *Os05g0553400* (*OsMYB55*; *LOC_Os05g48010*) was predicted as a candidate in the long arm of chromosome 5 through bulk segregation analysis (BSA) in our previous study [20]. This predicted gene was also identified as the potential candidate by RNA-seq and showed differential expression both in RNA-seq data and qPCR analysis with a 2.5-fold higher expression in *pl* mutant compared with WT (Figure 8). This higher relative expression levels of genes together with regulatory transcription factor genes might contribute to higher anthocyanin content in *pl* plants.

## 3. Discussion

Currently, purple color in plants has achieved much attention because of its advantageous biological functions. The distribution and amount of three types of colors (chlorophylls, carotenoids, and anthocyanins/flavonoids) are responsible for various pigments that are found in different plant tissues. Chlorophyll is higher than other types of pigments in green plants, whereas in yellow-leaved plants, carotenoid is higher; in purple-, red-, and blue-leaved plants, anthocyanin is higher than the other two pigments [21]. However, this insight is obviously lacking and still not well understood. For a better understanding of the underlying reason of leaf coloring, researchers are searching for mutants with changed phenotypes. A new rice mutant, *p1*, with a purple leaf color at the grain filling stage was identified [20], which confirmed that a purple leaf color in *pl* mutant was attributed due to a single recessive gene. The *p1* plants showed abnormal and disintegrated chloroplast cells with decreased chlorophyll content [20]. In this study, RNA-seq data showed variations in the transcriptome of *p1* mutant from the WT of rice plants at the grain filling stage. RNA-seq helps in the detailed transcriptomic profiling, annotation, and identification of genes [17,22,23,24].

The correlation coefficient of gene expression values among the tested samples reflects the consistency of the RNA-Seq data of the samples. The correlation coefficient among the *p1* samples was 0.98, whereas it was 0.96 in WT samples, indicating the consistency of the RNA-seq data among the samples because the values were in the vicinity of unity [18]. 

Confirmation of the expression of predicted DEGs through qRT-PCR is necessary for further functional analysis of the transcriptomes [18,25]. The qRT-PCR was conducted using 20 randomly selected genes, which were predicted as DEGs. GO analysis conferred that up-regulated DEGs are involved in phenylpropanoid biosynthesis process (Figure S1). Moreover, KEGG and pathway enrichment analyses showed the uni-genes involved in phenylpropanoid, phenylalanine metabolism, and flavonoid biosynthesis pathways as well as leaf coloration [8,21,26]. 

Anthocyanins are a group of flavonoids and are synthesized in the phenylpropanoid pathway, which has been studied thoroughly [26]. At the beginning of the flavonoid pathway, 4-coumaroyl CoA is catalyzed to naringenin chalcone to naringenin and to dihydroflavonol with the catalyzation by chalcone synthase (CHS), chalcone isomerase (CHI), and flavanone 3-hydroxylase (F3H), respectively. Thereafter, dihydrokaempferol (DHK) is hydroxylated by F3′H to produce dihydroquercetin, which is converted to leucocyanidin and then to cyanidin by DFR and ANS activities, respectively [9,27]. Finally, decorative anthocyanins are produced and transported to vacuole due to activities of transcription factors and transporter genes. The anthocyanin pathway has been genetically and biochemically explained in *Arabidopsis*. Due to knockout mutations of biosynthetic genes, less pro-anthocyanidin and anthocyanin is produced in *Arabidopsis* seeds [4,28,29]. Four genes encode the isomers of *PAL*, of which *PAL1* and *PAL2* are the preferred flavonoid pathways at nitrogen deficit and cold temperature conditions [28,30,31,32]. In our study, the DEGs of phenylpropanoid and flavonoid biosynthesis pathways were up-regulated, for example: *naregenin-chalcone synthase* (*CHS*)*, chalcone isomerase* (*CHI*), *flavonol synthase* (*FAS*) and *UDP-glucosyl transferase*. The orthologous of *PAL* family genes: *OsPAL1* (*Os02g0626100*), *OsPAL2* (*Os02g0626400*), *OsPAL4* (*Os02g0627100*), *OsPAL6* (*Os04g0518400*), and *OsPAL7* (*Os05g0427400*) were highly up-regulated in *pl* samples. In addition, *C4H* (*Os05g0320700*), *4CL* genes (*Os02g0177600* and *Os08g0448000*), *OsF3H* (*Os03g0122300*), anthocyanidin *5,3-O-glucosyltransferase* (*Os01g0734800*) were also up-regulated in *pl* samples (Table S3). 

Anthocyanins are synthesized due to the up-regulation of structural and transcription factor genes regulated by different regulatory genes. The downstream genes of anthocyanin biosynthesis are regulated by WBM complex (*WD40-bHLH-MYB*). The *R2R3-MYB* proteins contain two MYB domain, such as *MYB75*/*PAP1*, *MYB90*/*PAP2*, *MYB113* and *MYB114*, and are regulated by anthocyanin biosynthesis in vegetative tissues [28,33,34]. In plants, different tissue attains various levels of anthocyanin due to the degree of expression of *R2R3*-*MYB* transcription factors of MBW complex [35,36,37,38], consistent with our findings. Anthocyanin accumulation in apple and purple-head Chinese cabbage are regulated by *MdMYB1* and *BrMYB2*, respectively [39], and also the up-regulation of structural genes *BrF3’H*, *BrDFR1*, *BrANS1*, and *BrUGTs* [40]. In pear (*Pyrus pyrifolia*) fruit, light-induced anthocyanins are produced through the binding of *PpMYB10* to the promoters of *CHS* and *CHI* genes [41]. The roles of bHLH transcription factors and WD proteins are pleiotropic [42]. Simultaneous up-regulation of *MYB* and *bHLH* with structural genes in *Arabidopsis*, cauliflower, and petunia is essential for anthocyanin biosynthesis [9,35,42,43]. 

In red-skinned pear (*Pyrus comunis* L.), *PybHLH3* and *PyMYB114* transcription interact together and promote anthocyanin accumulation [44]. Anthocyanin biosynthesis in apple is modulated by the interaction of *MdMYB308L* and *MdbHLH33* through binding to *MdDFR* promoter [45].

**Figure 10:**
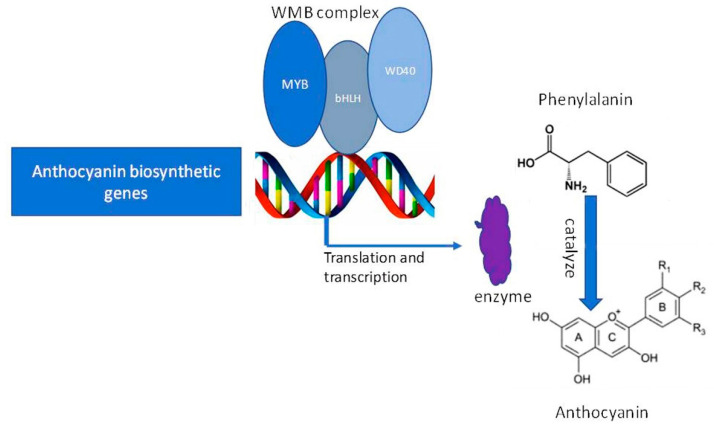
Proposed model for summarizing the transcriptional complex; MYB, bHLH, and WD40 proteins coordinately regulate anthocyanin accumulation through binding to the anthocyanin biosynthetic structural genes.

Two MYB genes ((*Os05g0553400* (*OsMYB55*) and *Os08g0428200*)), two bHLH family genes (*Os01g0196300* and *Os04g0300600*), and two WD40 family genes (*Os11g0132700* and *Os11g0610700*) showed up-regulation in *pl* mutant. In our previous study, we reported that *OsMYB55* (*LOC_Os05g48010*) with the ID *Os05g0553400* in RAP_locus might be the reason for accumulating anthocyanin in the *p1* mutant [20], consistent with other studies [35,46]. Our studies corroborated similar results of RNA-seq and qPCR expression analyses or BSA techniques for predicting candidate gene and identification. We narrate that the MYB, bHLH, and WD40 transcriptional complexes regulate phenylpropanoid metabolism through binding with flavonoid biosynthesis genes for enhancing anthocyanin accumulation (Figure 10). We speculate that R2R3-MYB transcription factors might be responsible for activating anthocyanin-biosynthesis genes, whereas, bHLH activates the transcription of MYB and consequently promotes anthocyanin accumulation.

Our RNA-seq data and the relative expression of the transcription factors showed up-regulation of two genes from each of the MYB, bHLH, and WD40 families are predicted to involve in anthocyanin accumulation through coordinated transcriptional activation of anthocyanin biosynthesis genes in *pl* mutant. Up-regulation of these three transcription factors might be influenced by the activity of the *OsMYB55* (*R2R3-MYB* transcription factor) coordinately in *p1* mutant for anthocyanin accumulation and purple coloration.

We profiled transcriptomes of *pl* vs. WT plants to figure out the transcripts involved in leaf coloration of rice. Through GO enrichment, KEGG pathway and KOG/COG analyses, we found that multiple pathways are intermingled in leaf coloration in rice. Based on the literature, we presumed that the phenylpropanoid pathway might contribute more than any other pathways to leaf coloration of rice. From our transcript data, we found that up-regulation of the members of transcriptional regulation complex (*MYB*, *bHLH*, and *WD40*) coordinately regulates anthocyanin accumulation. Among this complex, the *R2R3-MYB* transcription factors activate the biosynthetic genes and take part in leaf coloration. These results could give an advanced understanding of the leaf coloration process in rice and might also provide the experimental insight for manipulating purple color traits of rice through breeding programs. 

## 4. Materials and Experimental Methods

### 4.1. Plant Materials and Sample Collection

Flag leaves of cultivar Zhenong 34 and the *purple leaf* (*pl*) mutant of *Oryza sativa* L. ssp. *indica* were used for RNA sequencing. The *pl* mutant was induced through ethyl methane sulfonate (EMS) mutagenesis and it was identified phenotypically with purple coloration of leaf and leaf sheath at the grain filling stage in M_2_ population [20]. Ten flag leaves from each of the *pl* and WT plants at the grain filling stage were collected and pooled together. The pooled samples of *pl* and WT were immersed immediately in liquid nitrogen and stored in a freezer at −80 °C until RNA extraction.

### 4.2. Isolation of RNA, Library Construction and Sequencing

Total RNA was extracted using a total RNA extractor kit (Trizol) (B511311, Sangon, Shanghai, China) following the manufacturer’s instructions. The RNA quality and quantity were measured using agilent 2100 Bioanalyzer (Agilent Technologies, Blbd Santa Clara, CA, USA) and Nanodrop (IMPLEN, Westlake Village, CA, USA), respectively. High-grade RNA samples were sent to Sangon Biotech Co. Ltd. (Shanghai, China) for sequencing and further analyses. The mRNAs from total RNA were refined using poly-T oligo-attached magnetic bead. The first-strand cDNA was synthesized by using random hexamer primer and M-MuLV reverse transcriptase (RNase H-) and second-strand cDNA was synthesized by using DNA polymerase I and RNase H. Exonuclease/polymerase was used to change the remaining overhangs into blunted ends and the adaptors were ligated for preparing the libraries after adenylation at 3′ends of DNA fragments. Forward primer: 5′ AGATCGGAAGAGCACACGTCTGAAC 3’ and reverse primer: 5′ AGATCGGAAGAGCGTCGTGTAGGGA 3′ were used in preparing the libraries. A total of six libraries—three from each of the *pl* mutant and WT—were produced and Illumina HiSeqTM2000 (Illumina, San Diego, CA, USA) was used for paired-end sequencing of the constructed libraries.

### 4.3. RNA-Seq Data Assay

Raw reads gathered from Illumina HiSeqTM 2000 sequencing were trimmed to retrieve the clear reads. Adapter, poly-N, and poor-quality reads were eliminated during purification of the data to improve the accuracy of the analysis. The raw reads were cleaned by FASTQ [18,47]. The cleaned and purified reads were kept in FASTQ format and were mapped to the reference gene using hierarchical indexing for spliced alignment of transcripts (Hisat2; version 2.1.0) with default parameters [48]. The raw sequences are uploaded in the NCBI under accession no. prjna760250 (https://www.ncbi.nlm.nih.gov/bioproject/?term=prjna760250/, accessed on 10 September 2021). Nipponbare genome (http://rice.plantbiology.msu.edu/, accessed on 12 October 2020) was used as reference to identify the perfect match and mismatch [49]. For defining the transcript isoforms of the similar gene, RNA-seq by expectation maximization (RSEM) was employed [50]. Whole RNA-seq was conducted using the pipeline described in Figure S2.

### 4.4. Transcriptomic Analysis 

DESeq2 (version 1.12.4) was utilized for identifying the DEGs from the samples [51]. Genes were considered as differentially expressed, where q < 0.001 and log_2_|fold change| > 2. Here, q is the quality factor used to control the false-positive rate whilst carrying out multiple tests, and the fold change showed the multiple of expression difference. Strict filtering was applied via independent filtering for removal of the expression values which were equal to zero or NA on the mean of normalized counts and plotted on log plot. The transcript per million (TPM) was used to normalize the reads and was expressed as fragments per kilo base per million (FPKM).

DEGs were assigned to GO terms (biological functions) in the database using ClusterProfiler [52] and a hypergeometric assessment was carried out for identifying GO enriched for the genes from the reference genome. KEGG pathway and the DEGs were compared to reference genome for identifying significantly enriched metabolic pathways. 

### 4.5. qRT PCR Analysis

For validation of RNA-sequencing data, a total of 20 genes were selected randomly from DEGs and determined the expression level using qRT PCR (quantitative real-time PCR). Primer 5 software version 5 was used for designing the gene-specific primers (Table S11). The qRT-PCR was conducted with 10 μL reaction mixture using Ex TaqII (Takara, Tokyo, Japan) in a Roche light cycler 480 (Roche, Stuttgart, Germany) and the reaction was repeated five times as replication. The reaction mixture contained 1 µL template cDNA (50 ng), 2 µL ddH_2_O, 5 µL 2× SYBR mix, and 1 µL (10 pmol) of each forward (F) and reverse (R) primers. The qRT-PCR was performed using the thermal protocol; denaturation 95 °C for 30 s; thereafter, 40 cycles of denaturation; 95 °C for 5 s, annealing; 55 °C for 20 s and extension; 72 °C for 10 s. The *OsActin* gene was used as an internal control for calculating the relative expression [53]. For calculating the relative gene expression, cq values were normalized through cq of *OsActin* following the 2^−ΔΔCT^ method [54]. A significance test for mean differences of gene expression was performed for 5 repeats between *pl* and WT plants per gene following a Tukey test (*p ≤* 0.05) using Minitab 18 (State College, Borough, PA, USA). 

### 4.6. Anthocyanin Content Measurement

Total anthocyanin in leaf and leaf sheath of *pl* and WT was estimated by using spectro-phtotometer UV-1800 (Shanghai, China) following the protocol [8]. Frozen leaf samples were grinded in a mortar and pestle placed inside liquid nitrogen and anthocyanin was extracted using acidic alcohol (1% HCl and 95% ethanol). Total anthocyanin was measured as: anthocyanins = (A530 − 0.25 × A657) × FW − 1; here, A530 = absorbance at 530 nm, A657 = absorbance at 657 nm, and FW = weight of fresh leaf (g). Each sample was repeated 3 times in quantification.

## Figures and Tables

**Table 1:** Summary of reads of the samples retrieved from Illumina sequencing.

Sample Name	Raw Reads	Average Raw Read Length (bp)	Clean Reads	Average Clean Read Length (bp)	Error (%)	Q20 (%)	Q30 (%)	GC Content (%)
WT	55,160,750	150	53,875,169	144.14	0.00	99.20	97.06	57.83
*pl*	54,237,834	150	52,941,300	142.99	0.00	99.18	97.03	57.71

**Table 2:** Statistical summary of alignment results of RNA-seq used in mapping.

Sample Name	Total Clean Reads in Mapping	Clean Read Mapped (%)	Multiple Mapped (%)	Uniquely Mapped (%)	Non-Splice Reads (%)	Splice Reads (%)	Reads Mapped in Proper Pairs (%)
WT	45,766,956	84.95	2.74	82.21	55.13	27.08	81.31
*pl*	45,900,107	86.70	6.06	77.99	51.72	26.28	83.06

**Table 3:** Relative gene expression analysis of 20 randomly selected genes for making the comparison of *pl* and WT group conducted by RNA- seq and qRT PCR.

Gene ID	Gene Name *	*pl* vs. WT		Gene Description	Pathway/Function Involved
		RNA-seq log_2_ fold change	qRT PCR fold change		
*Os02g0626100*	*OsPAL1*	3.19286832	3.01 ± 0.01	Similar to Phenylalanine ammonia-lyase	Anthocyanin Biosynthesis
*Os02g0626400*	*OsPAL2*	2.10996231	1.93 ± 0.03	Phenylalanine ammonia-lyase	Phenylpropanoid metabolism, Anthocyanin Biosynthesis
*Os04g0518400*	*OsPAL6*	5.02738052	3.10 ± 0.19	Similar to Phenylalanine ammonia-lyase	Phenylpropanoid metabolism, Anthocyanin Biosynthesis
*Os05g0427400*	*OsPAL7*	3.03883343	1.25 ± 0.10	Similar to Phenylalanine ammonia-lyase	Phenylpropanoid metabolism
*Os02g0177600*	*Os4CL3*	1.82828648	1.19 ± 0.10	4-coumarate: coenzyme A ligase	Phenylpropanoid metabolism
*Os08g0448000*	*Os4CL5*	4.87114854	2.08 ± 0.14	4-coumarate: coenzyme A ligase 5	Phenylpropanoid metabolism
*Os03g0122300*	*OsF3H*	4.76468487	2.37 ± 0.24	flavonol synthase	Flavonoid biosynthesis, Anthocyanin Biosynthesis
*Os08g0441500*		4.4886503	3.93 ± 0.015	cinnamoyl-CoA reductase	Phenylpropanoid metabolism, Anthocyanin Biosynthesis
*Os05g0320700*		1.8282864	1.65 ± 0.31	cinnamate 4-hydroxylase 2	Phenylpropanoid metabolism, Anthocyanin Biosynthesis
*Os10g0512400*	*OsCAld5H1*; *F5H1*	3.1612775	2.57 ± 0.15	coniferaldehyde 5-hydroxylase 1, ferulate 5-hydroxylase 1	Lignin biosynthesis
*Os01g0196300*	*DPF*; *OsbHLH025*	5.6010349	3.65 ± 0.020	basic helix-loop-helix protein 025	
*Os01g0838350*	*Phosphate-limitation inducible gene 1*	−1.0446037	−0.59 ± 0.09	Conserved hypothetical protein	
*Os01g0734800*		1.120071	1.65 ± 0.10	anthocyanidin 5,3-O-glucosyltransferase	Anthocyanin Biosynthesis
*Os11g0116300*		2.70218828	2.25 ± 0.02	chalcone isomerase	Anthocyanin Biosynthesis
*Os02g0611800*		2.38197885	1.26 ± 0.08	Hydroxycinnamoyltransferase 2	Phenylpropanoid and Flavonoid biosynthesis
*Os02g0194700*	*OsLOX1*	−2.9658063	−0.68 ± 0.06	Lipoxygenase 1	
*Os11g0610700*	*OsWD40-190*	1.2940596	1.71 ± 0.10	WD40 repeat-like domain containing protein	
*Os07g0543100*		−2.9658063	−0.30 ± 0.01	Beta-amylase 1	
*Os02g0738100*		3.24756852	1.19 ± 0.13	basic helix-loop-helix protein	
*Os04g0662600*		4.492093	1.66 ± 0.07	Flavanone 3-hydroxylase 1	Flavonoid biosynthesis

* Gene IDs are arranged following the gene order of the anthocyanin biosynthetic pathway.

## Data Availability

All data will be available in the NCBI accession PRJNA760250 along with this manuscript upon publication.

## Supplementary Materials

The following are available online at https://www.mdpi.com/article/10.3390/ijms22189787/s1.
