# Supplementary material for: RNA-Seq-Based Profiling of pl Mutant Reveals Transcriptional Regulation of Anthocyanin Biosynthesis in Rice (Oryza sativa L.)
Source: Int J Mol Sci. 2021 Sep 10;22(18):9787. doi: 10.3390/ijms22189787 (PMC8466560; doi:10.3390/ijms22189787)
Supplement: Supplementary file 1 [file ijms-22-09787-s001.zip › Supplemental Figure.pptx]

## Slide 1
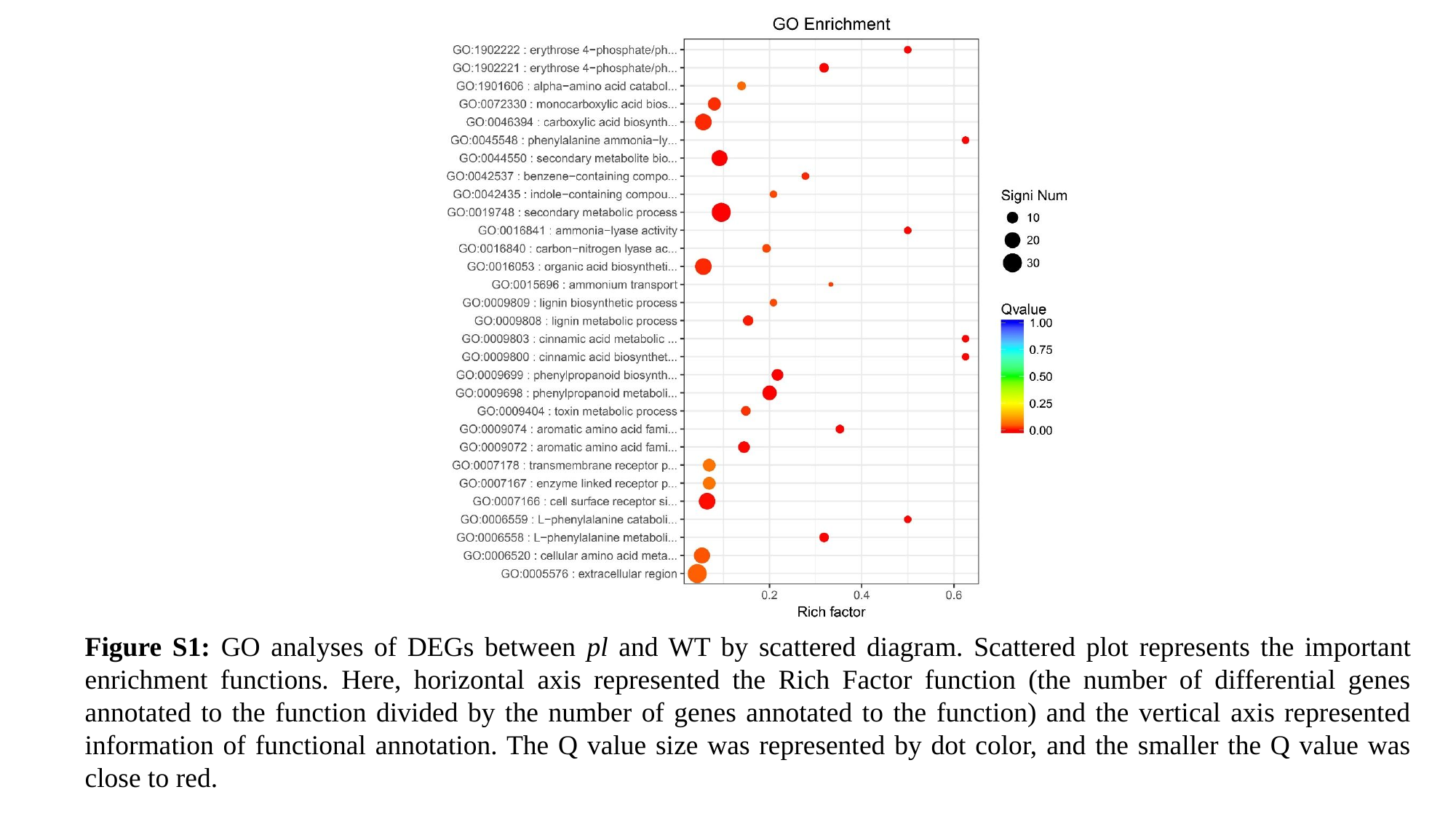

Figure S1: GO analyses of DEGs between pl and WT by scattered diagram. Scattered plot represents the important enrichment functions. Here, horizontal axis represented the Rich Factor function (the number of differential genes annotated to the function divided by the number of genes annotated to the function) and the vertical axis represented information of functional annotation. The Q value size was represented by dot color, and the smaller the Q value was close to red.

## Slide 2
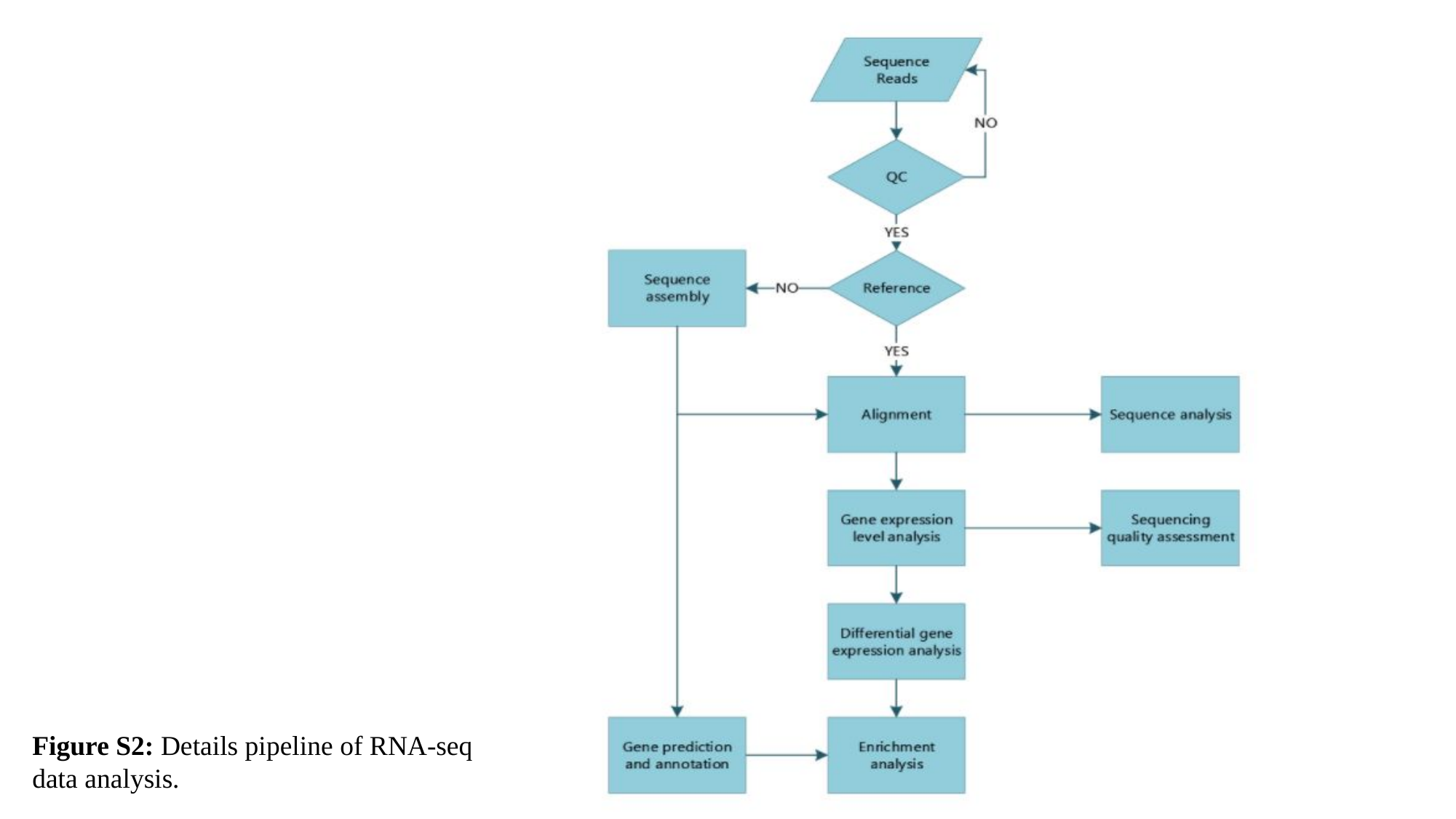

Figure S2: Details pipeline of RNA-seq data analysis.
