# Supplementary material for: RNA-Seq-Based Profiling of pl Mutant Reveals Transcriptional Regulation of Anthocyanin Biosynthesis in Rice (Oryza sativa L.)
Source: Int J Mol Sci. 2021 Sep 10;22(18):9787. doi: 10.3390/ijms22189787 (PMC8466560; doi:10.3390/ijms22189787)
Supplement: Supplementary file 1 [file ijms-22-09787-s001.zip › Table S1 & Table S2.pdf]

Table S1. Summary of total SNPs in WT and *pl* compared with the reference genome

	WT	*pl*
Total SNP (No.)	58361	59358
Total INDEL (No.)	5297	5648
SNP_effect_region		
Intergenic region (No.)	8195	8451
5-UTR (No.)	9000	8526
CDS (No.)	49355	48103
Intronic (No.)	2676	2487
ncRNA (No.)	1341	1347
3-UTR (No.)	16717	16540
Splice (No.)	1138	1135

Table S2. Types of nucleotide substitutions in detected homozygote polymorphic SNPs between WT and *pl*

Types of nucleotide substitution	WT	*pl*
Transitions		
A->G	9029	9366
C->T	9816	10032
G->A	9803	9956
T->C	9033	9363
Transversions		
A->C	2397	2560
A->T	1762	1872
C->A	3692	3251
C->G	2542	2688
G->C	2555	2700
G->T	3669	3200
