# Supplementary material for: RNA-Seq-Based Profiling of pl Mutant Reveals Transcriptional Regulation of Anthocyanin Biosynthesis in Rice (Oryza sativa L.)
Source: Int J Mol Sci. 2021 Sep 10;22(18):9787. doi: 10.3390/ijms22189787 (PMC8466560; doi:10.3390/ijms22189787)
Supplement: Supplementary file 1 [file ijms-22-09787-s001.zip › Table S10- Table S11.pdf]

Table S10. Total anthocyanin content (umole g-1 FW) of leaf and leaf sheath of *pl* mutant and WT samples

	WT	*pl*
Leaf	0.155 ± 0.01	0.405 ± 0.03
Sheath	0.112± 0.02	0.912 ± 0.06

Table S11. List of the primers used in this study for qRT-PCR

Gene ID	Forward primer	Reverse primer
Os02g0626100	*CGGTCGTTCCCGCTCTAC*	*TCGCCGTTCCACTCCTT*
Os02g0626400	*ACCGCCTCCGGTGACT*	*CGCCCTTGAACCCATAG*
Os04g0518400	*CTCTGCTCCAGGGGTACTCT*	*GCTTCTTCGCCAGCATCATG*
Os05g0427400	*CACATCTTGGAAGGCAG*	*GAGAGGTTGGAGGGCAG*
Os02g0177600	*ACGACGAGATCTTCATCGT*	*TTGTAGAACACCACCTCTTT*
Os08g0448000	CGAGAAGACGATCGACAAGGA	TCAGCACGCCGGAATTCCAGCA
Os03g0122300	CGCTACCTCCCTGATTGG	ACCTTCTTGATGTAGTCCT
Os08g0441500	*TTCCACACCGCCTCCCC*	*GATGGCAATCCCGCCCTG*
Os05g0320700	*ATCCCGCTGCTGGTGCC*	*GGCGTGCTTGAGGATCT*
Os10g0512400	*TTCAACCTCACCAAGAAC*	*TCTTGTCGATGAACCTGTC*
Os01g0196300	*CGTGCAAACCTAACATTACA*	*GGCACCTCCCTTTTTCTTCTT*
Os01g0838350	TGTAGGGCAACTAAACAAC	TGACAAATACTAGTTCACCA
Os01g0734800	GTGTTCTCCGCGGAGCAGCT	TTCTCCACATCGGACAGGAA
Os11g0116300	*ATGTACTGTGCAGTGGGCACA*	*TCATGCAGAGAGCAGTGCTGC*
Os02g0611800	*ATGAAGATCAACGTGCGG*	*CCGCACGTTGATCTTCAT*
Os02g0194700	*ATCCTGTCGTCGCACTCC*	*AGCTCGTACGGCACGAT*
Os11g0610700	*CAATCCGCCTCCTCCGGT*	*AGCCCTCAAAGCTATATTA*
Os07g0543100	*TCAACTTCACCTGCGCC*	*TTCCTGACGAAGGCCTTGAA*
Os02g0738100	*CACAGGCTACCACCGTT*	*ATGAGTATAGAGCTGGGACTT*
Os04g0662600	AACTTCTACCCCAAGTGCCCC	GTCGAGAGACTTGGGCTTCT
Actin	*GTGGTCGCCCCTCCTGAAAG*	*GGCTTAGCATTCTTGGGTCCG*
